# Pulmonary vein isolation using cryoballoon technique in atrial fibrillation patient after Greenfield vena cava filter implantation

**DOI:** 10.21542/gcsp.2020.21

**Published:** 2020-11-30

**Authors:** Shaojie Chen, Boris Schmidt, Stefano Bordignon, Shota Tohoku, K.R. Julian Chun

**Affiliations:** 1 Cardioangiologisches Centrum Bethanien (CCB), Frankfurt Academy For Arrhythmias (FAFA), Kardiologie, Medizinische Klinik III, Agaplesion Markus Krankenhaus, Frankfurt am Main, Germany; 2 Die Sektion Medizin, Universität zu Lübeck, Lübeck, Germany

## Abstract

**Background:** Cryoballoon ablation is an established procedure for atrial fibrillation (AF). Patient with vena cava filter undergoing pulmonary vein isolation (PVI) were seldom reported.

**Case presentation:** We describe an AF ablation technique using the second generation cryoballoon in a patient after vena cava filter implantation. All pulmonary veins were successfully isolated without complication.

**Conclusions:** For AF patient with previously implanted vena cava filter, cryoballoon based PVI appears feasible and safe.

## Introduction

Catheter ablation is an effective therapeutic option in treating patients with atrial fibrillation, pulmonary vein isolation (PVI) remains the cornerstone ablation strategy (AF) regardless the classification of the AF^1^. Cryoballoon ablation is an established procedure for patents with symptomatic atrial fibrillation (AF).

An inferior vena cava filter is a medical device implanted into the inferior vena cava to prevent pulmonary emboli in patient with venous thromboembolism. Patients who had previous vena cava filter undergoing PVI were seldom reported. The major concern for such clinical scenario is mechanic dislodgement of the filter device during the ablation procedure.

### Case

A 76 years old female patient was admitted to our center because of symptomatic drug-refractory persistent atrial fibrillation (AF). The CHA2DS2-VASc score was 4 and HAS-BLED score was 3. A Greenfield-Filter was previously implanted due to venous thromboembolism and recurrent pulmonary emboli.

**Figure 1:**
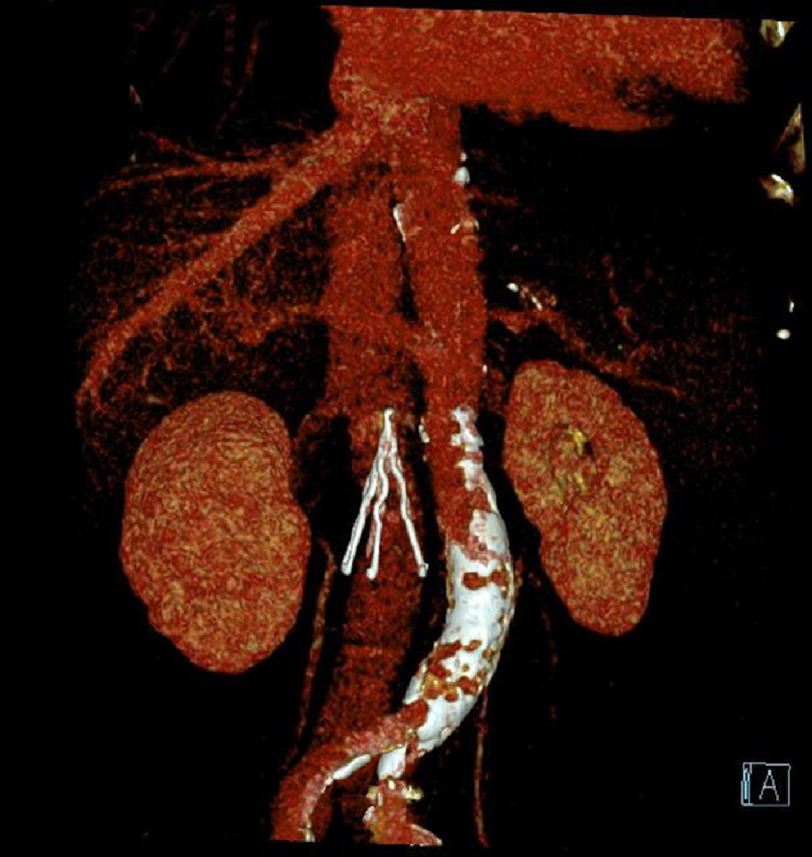
CT shows inferior vena cava filter.

Abdominal computed tomography (CT) scan was performed; no device-related thrombus was detected (Figure 1). Intracardiac thrombus was ruled out by transesophageal echocardiography (TEE). After fully discussion, we decided to perform AF ablation using the cryoballoon (CB) technology. Full consent of the patient was obtained before the procedure.

**Figure 2:**
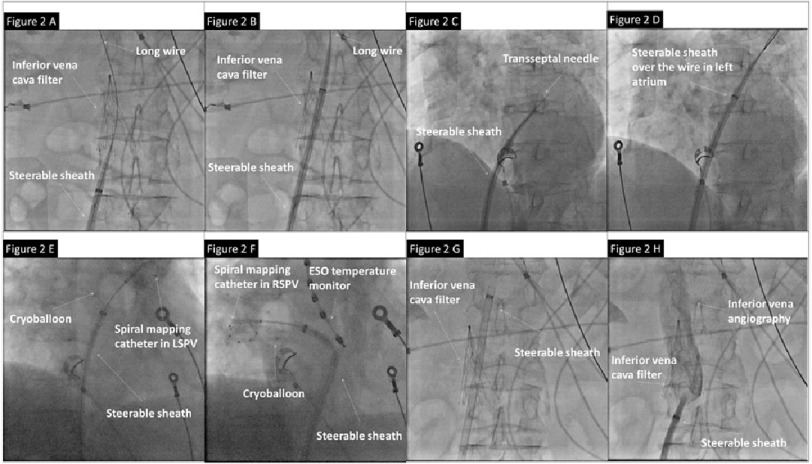
CB PVI in patient with inferior vena cava filter.

The institutional approach of CB ablation was published previously^2–4^. The key procedural techniques were detailed in Figure 2. In this case, after single groin puncture, the CB steerable sheath (12F, Flex Cath Advance, Medtronic) was carefully advanced into the right superior vena cava through the Greenfield Filter using “over the wire” technique. After single transseptal puncture using the modified Brockenbrough technique (BRK-1 needle, St Jude Medical; Flex Cath Advance steerable sheath, Medtronic), selective PV angiography was performed to identify the pulmonary veins. A second-generation cryoballoon (CB 2, Arctic Front Advance, Medtronic) was utilized for PVI. All four PVs were successfully isolated with time-to-effect guided freeze approach^5^. By the end of the procedure, the cryoballoon and steerable sheath were safely withdrawn without dislodgement of the Greenfield-Filter device.

The patient was scheduled for outpatient clinic visit at 3, 6, 12 months after the procedure. The Follow-up assessments showed favorable outcome without clinical AF/AT recurrence.

### What have we learned?

An inferior vena cava filter is a medical device implanted into the inferior vena cava to prevent pulmonary emboli in patient with venous thromboembolism. Patients who had previous vena cava filter undergoing catheter ablation for atrial fibrillation were seldom reported. The major concern for such clinical scenario is mechanic dislodgement of the filter device during the ablation procedure. This report shows that for AF patient with previously implanted vena cava filter, a step-by-step approach based cryoballoon ablation appears feasible and safe.

## Conflict of interest

None.

## References

[1] Chen S, Pürerfellner H, Meyer C, Acou WJ, Schratter A, Ling Z, Liu S, Yin Y, Martinek M, Kiuchi MG, Schmidt B, Chun KRJ (2019a). Rhythm control for patients with atrial fibrillation complicated with heart failure in the contemporary era of catheter ablation: a stratified pooled analysis of randomized data. European Heart Journal.

[3] Chen S, Schmidt B, Bordignon S, Bologna F, Perrotta L, Nagase T, Chun KRJ (2018b). Atrial fibrillation ablation using cryoballoon technology: Recent advances and practical techniques. J Cardiovasc Electrophysiol.

[2] Chen S, Schmidt B, Bordignon S, Bologna F, Nagase T, Perrotta L, Julian Chun KR (2018a). Practical Techniques in Cryoballoon Ablation: How to Isolate Inferior Pulmonary Veins. Arrhythm Electrophysiol Rev.

[4] Chen S, Schmidt B, Bordignon S, Perrotta L, Bologna F, Chun KRJ (2019b). Impact of Cryoballoon Freeze Duration on Long-Term Durability of Pulmonary Vein Isolation: ICE Re-Map Study. JACC Clin Electrophysiol.

[5] Chun KR, Stich M, Fürnkranz A, Bordignon S, Perrotta L, Dugo D, Bologna F, Schmidt B (2017). Individualized cryoballoon energy pulmonary vein isolation guided by real-time pulmonary vein recordings, the randomized ICE-T trial. Heart Rhythm.

